# Inflammatory Mediators and Type 2 Diabetes Risk Factors before and in Response to Lifestyle Intervention among Latino Adolescents with Obesity

**DOI:** 10.3390/nu15112442

**Published:** 2023-05-24

**Authors:** Armando Peña, Micah L. Olson, Stephanie L. Ayers, Dorothy D. Sears, Sonia Vega-López, Abigail T. Colburn, Gabriel Q. Shaibi

**Affiliations:** 1 Department of Health and Wellness Design, School of Public Health-Bloomington, Indiana University, Bloomington, IN 47405, USA; 2 College of Health Solutions, Arizona State University, Phoenix, AZ 85004, USA; dorothy.sears@asu.edu (D.D.S.);; 3 Center for Health Promotion and Disease Prevention, Arizona State University, Phoenix, AZ 85004, USA; molson@phoenixchildrens.com (M.L.O.);; 4 Division of Pediatric Endocrinology and Diabetes, Phoenix Children’s Hospital, Phoenix, AZ 85016, USA; 5 Southwestern Interdisciplinary Research Center, Arizona State University, Phoenix, AZ 85004, USA; stephanie.l.ayers@asu.edu; 6 Obstetrics, Gynecology and Reproductive Sciences, Yale School of Medicine, New Haven, CT 06510, USA; 7 John B. Pierce Laboratory, Yale School of Medicine, New Haven, CT 06519, USA

**Keywords:** exercise, nutrition, pediatric obesity, diabetes prevention, adipokine, inflammation

## Abstract

Obesity is associated with chronic inflammation that may contribute to T2D among youth. We examined the association between inflammatory biomarkers and insulin sensitivity and β-cell function and response to lifestyle intervention among Latino youth with obesity. Latino youth (n = 64) were randomized to six months of lifestyle intervention (INT, n = 40) or usual care (UC, n = 24). INT included nutrition education and physical activity. UC involved meeting with a pediatric endocrinologist and registered dietitian to discuss healthy lifestyles. At baseline, multiple linear regression assessed fasting serum interleukin-6 (IL-6), tumor necrosis factor-alpha (TNF-α), monocyte chemoattractant protein-1 (MCP-1), high-molecular weight adiponectin (HMW Adpn), IL-10, IL-1 receptor antagonist (IL-1ra) as predictors of insulin sensitivity (whole-body insulin sensitivity index, WBISI) and β-cell function (oral disposition index, oDI). Changes in outcomes between groups were assessed using covariance pattern models. At baseline, MCP-1 (β ± SE, −0.12 ± 0.05, *p* = 0.027) and IL-1ra (−0.03 ± 0.01, *p* = 0.005) were negatively associated with WBISI. Treatment effects were not observed for inflammatory markers. WBISI was significantly increased among both INT (from 1.8 ± 0.2 to 2.6 ± 0.4, *p* = 0.005) and UC (from 1.6 ± 0.2 to 2.8 ± 0.5, *p* = 0.002) with no significant differences between the groups. Obesity-related inflammatory mediators were associated with T2D risk factors but were unaffected by lifestyle intervention among Latino youth.

## 1. Introduction

Pediatric obesity is characterized by chronic inflammation, which has been described as an increase in pro-inflammatory mediators and decreased anti-inflammatory mediators [1]. Chronic inflammation is associated with decreased insulin sensitivity (i.e., insulin resistance) and pancreatic β-cell dysfunction [2,3], which are the two central pathophysiologic processes underpinning T2D. Lifestyle intervention is the cornerstone approach for preventing T2D among high-risk adults and it has demonstrated favorable effects on pro- and anti-inflammatory mediators [4,5]. However, gaps remain in the pediatric literature pertaining to lifestyle intervention effects on pro- and anti-inflammatory mediators in high-risk Latino youth populations.

In obesity, immune cells infiltrate adipose tissue and produce pro-inflammatory mediators, including cytokines and chemokines [1]. Interleukin-6 (IL-6) and tumor necrosis factor-alpha (TNF-α) are cytokines activated by pro-inflammatory signaling pathways in adipose tissue macrophages and are elevated in obesity [6,7,8]. Monocyte chemoattractant protein-1 (MCP-1) is a chemokine upregulated by pro-inflammatory macrophage pathways which facilitates the infiltration of pro-inflammatory immune cells in adipose tissue [9]. IL-6, TNF-α, and MCP-1 have been associated with impairments in insulin sensitivity and β-cell function [2,10,11,12,13]. Adipose tissue also produces anti-inflammatory mediators, including adiponectin (Adpn), interleukin-10 (IL-10), and interleukin-1ra (IL-1ra), all of which have been shown to exert insulin sensitizing and β-cell protecting effects [14,15,16]. Studies have demonstrated significantly lower Adpn and IL-10 concentrations among youth with obesity compared to normal weight, potentially hampering the defense mechanisms against inflammation [17,18]. Interestingly, IL-1ra is significantly increased in pediatric obesity [19,20], which may be due to its upregulation by pro-inflammatory pathways [21]. Taken together, obesity is associated with non-homeostatic levels of key inflammatory mediators that serve as candidate physiologic targets to improve insulin action and glucose metabolism.

Comprehensive lifestyle interventions target changes in physical activity and eating behaviors to improve health. Physical activity has been traditionally studied in the form of exercise. Acute exercise has been associated with increases in anti-inflammatory mediators IL-6 and IL-1ra [17,22], while exercise interventions have demonstrated favorable effects on upstream inflammatory markers [23,24,25,26]. Consuming unsaturated fat, fruits, and vegetables can also promote health and have anti-inflammatory properties [27,28]. Studies have demonstrated that omega-3 polyunsaturated fatty acids downregulate pro-inflammatory pathways (e.g., nuclear factor kappa-light-chain-enhancer of activated B cells pathway) and upregulate anti-inflammatory markers (e.g., peroxisome proliferator antagonist receptor-alpha and -gamma) [29]. Furthermore, phytochemicals found in fruits and vegetables have been shown to induce shifts in macrophage phenotypes from pro-inflammatory to anti-inflammatory phenotypes [30]. Thus, it stands to reason that lifestyle interventions that promote physical activity and healthy eating behaviors have the potential to modify pro- and anti-inflammatory mediators to homeostatic levels among youth with obesity. Lifestyle intervention studies have demonstrated favorable results on inflammatory mediators among high-risk youth; however, this literature predominantly focuses on adiponectin and pays less attention to other key obesity-related inflammatory mediators that have emerged as relevant to the pathophysiology of T2D, including insulin sensitivity and β-cell function [18,31,32,33,34].

Understanding the associations between key pro- and anti-inflammatory mediators with T2D risk factors and how they are impacted by lifestyle intervention may shed light on the mechanisms by which healthy lifestyle behaviors can prevent or delay T2D. Therefore, the purposes of this study were (1) to examine the associations of obesity-related pro- and anti-inflammatory mediators (IL-6, TNF-α, MCP-1, Adpn, IL-10, and IL-1ra) with insulin sensitivity and β-cell function in high-risk Latino youth and (2) to examine the response of these inflammatory mediators to lifestyle intervention compared to usual care among high-risk Latino youth.

## 2. Materials and Methods

*Participants.* Latino youth with obesity and prediabetes were recruited and enrolled as part of a randomized control trial [35]. Specific inclusion criteria were as follows: (1) self-reported Latino descent, (2) ages 12–16 years, (3) BMI% > 95th for age and sex, and (4) prediabetes using the criteria set forth by the American Diabetes Association as defined by HbA1c 5.7–6.4%, fasting glucose 100–125 mg/dL, or an expanded definition for 2-h glucose, 120–199 mg/dL, following a 75 g oral glucose tolerance test (OGTT) [36]. Youth were excluded if they (1) were taking medication(s) or diagnosed with a condition that influences carbohydrate metabolism, physical activity, and/or cognition, (2) met the criteria for diabetes (fasting glucose ≥ 126 mg/dL, HbA1c ≥ 6.5%, or 2 h glucose ≥ 200 mg/dL), (3) were recently hospitalized (within previous two months), (4) are currently enrolled in (or within 6 months) a formal weight loss program, or (5) have an uncontrolled mental health condition. Youth and their parents provided written consent prior to their study participation. Spanish materials were available. Additionally, stored and consented serum samples allowed for the assessment of inflammatory biomarkers and set the basis for the present study. We included all youth who had no missing data at T1 for all variables of interest, including inflammatory mediators, body composition, and diabetes risk factors. The N for each measurement at T2 less than the full data set is noted in its respective table below. One participant’s data was excluded from analysis since Adpn concentrations at baseline (13.6 μg/mL; intervention group) were 232% higher than the second highest data point (4.1 μg/mL) in the dataset. This study included a total of 64 Latino youth and was approved by the Arizona State University (ASU) Institutional Review Board and is in accordance with the Declaration of Helsinki. 

*Research Design.* This was an ancillary study that stemmed from a two-arm parallel RCT testing the efficacy of a six-month diabetes prevention lifestyle intervention (INT) against a usual care (UC) condition on T2D risk factors [35]. Data collected at baseline (T1) and at six months (T2) were used for the current analysis. 

*Baseline and End Point Study Visits.* Participants arrived at the ASU clinical research unit following an overnight fast for assessment of height, weight, BMI, BMI-z, waist circumference, total body composition by dual x-ray absorptiometry (DEXA, Lunar iDXA, GE Healthcare, Chicago, IL, USA), and T2D risk factors from an oral glucose tolerance test (OGTT). Fasting blood was collected and serum were isolated and stored at −80 °C for further analysis of inflammatory markers. Insulin sensitivity and glucose tolerance were measured via a multiple sample 2 h 75 g OGTT with plasma samples collected at fasting and every 30 min for measurement of insulin and glucose concentrations.

*Biomarker Assessment*. Concentration of inflammatory markers IL-6, MCP-1, IL-10, and IL-1ra were measured in serum using a multiplex immunoassay (#K15067L-2; Meso Scale Discovery, Rockville, MD, USA). TNF-α was analyzed in serum using a single-plex immunoassay (#K151UCK-1; Meso Scale Discovery, Rockville, MD, USA). High-molecular weight adiponectin (HMW Adpn) is the most biologically relevant for insulin sensitivity (compared to low- and moderate-molecular weight Adpn) [37] and was measured in serum using an enzyme-linked immunosorbent assay, or ELISA (#80-ADPHU-E01: ALPCO, Salem, NH). Insulin was analyzed using ELISA (ALPCO, Salem, NH, USA) and glucose concentrations were analyzed photometrically via glucose oxidation (Cobas c111 analyzer, Roche Diagnostics, Indianapolis, IN, USA).

Insulin sensitivity was estimated using the whole-body insulin sensitivity index (WBISI). The WBISI was generated from insulin (ALPCO, Salem, NH, USA) and glucose (cobas c111 analyzer, Roche Diagnostics, Indianapolis, IN, USA) concentrations were measured in plasma collected during the OGTT. WBISI has been validated among youth with obesity [38]. Fasting, 30′, 60′, 90′, and 120′ insulin and glucose concentrations are inserted into a formula, $\frac{10,000}{\surd I_{0}\times G_{0}\times Mean\left(I_{0},I_{30},I_{60},I_{90},I_{120}\right)\times Mean\left(G_{0},G_{30},G_{60},G_{90},G_{120}\right)}$, to generate a WBISI score where higher scores correspond to increased insulin sensitivity levels. β-cell function was estimated by the oral disposition index (oDI), which is the product of insulin sensitivity (WBISI) and insulin secretion [39]. Insulin secretion was estimated by the insulinogenic index (IGI), which is a ratio of the difference in glucose and the difference in insulin in the first 30 min of the OGTT (IGI = ΔG_30_ − G_0_/ΔI_30_ − I_0_). Therefore, oDI was estimated as WBISI × IGI. Glucose tolerance was measured by 2 h post-challenge glucose concentrations during OGTT. Body composition assessment included total fat mass, total lean mass, and body fat percent measured using DEXA (Luna iDXA, GE Healthcare, Chicago, IL, USA).

*Intervention Group-Lifestyle*. The lifestyle intervention has been previously described in detail [35,40]. The lifestyle intervention included 1 day/week (75 min per session) of nutrition and health education with behavior change skills training and 3 days/week (60 min per session) of physical activity. Health education sessions were delivered by community health educators from a local community clinic to groups of 8–10 families and they promoted the adoption of a healthy balanced diet, including reducing saturated fat intake, added sugars, and sugar-sweetened beverages, managing portion sizes, and increasing intake of fiber, fruit, and vegetables. Physical activity sessions were delivered at the local YMCA with qualified instructors twice per week for 60 min/session. The physical activity curriculum included circuit training, sports activities (e.g., basketball, and soccer), agility and cardiovascular exercises so that average target heart rates per session were ≥150 beats per minute. A third day of physical activity was promoted and monitored by instructors on a weekly basis to complete a minimum of 180 min of moderate-to-vigorous physical activity per week. In-depth information regarding the lifestyle intervention, including nutrition education and physical activity sessions, can be found in Soltero et al. [35].

*Usual Care (UC) Group*. Participants randomized to UC met with a pediatric endocrinologist and registered dietitian on two occasions within the 6-month period from T1 to T2 to discuss laboratory results and develop SMART goals for making healthy lifestyle changes. The UC group was offered an abridged version of the lifestyle intervention after completion of the study.

*Analytical Approach.* Baseline characteristics between groups were compared using independent samples t-tests (continuous variables) and chi-square tests (categorical variables) with IBM SPSS 28.0.1 (Chicago, IL, USA). Baseline correlations were analyzed using the two-tailed Pearson correlation coefficient. In order to assess the associations between pro- and anti-inflammatory markers and insulin sensitivity and β-cell function at baseline, multiple linear regressions that use robust maximum likelihood estimation methods were conducted with the T2D risk factor as the dependent variable and all inflammatory markers included in each model as predictors. Age, sex, and fat mass were included as covariates in the multiple linear regression models. Changes in outcomes were compared between the groups using covariance pattern models in Mplus 8.7 (Los Angeles, CA, USA) which assess the difference in changes in outcomes from T1 to T2. Full information maximum likelihood (FIML) was used to account for missing data. Alpha level for all analyses are set at 0.05. Data are presented as mean ± SD, FIML-adjusted mean ± SE, or FIML-adjusted ΔMean ± 95%CI when appropriate.

## 3. Results

Baseline characteristics are described in Table 1. There were no significant differences in the inflammatory markers between INT and UC groups.

Bivariate associations between inflammatory markers and T2D risk factors are displayed in Supplementary Table S1. IL-6 was significantly positively associated with BMI and BMI-z, while TNF-α was positively associated with BMI-z. IL-1ra was significantly associated with all measures of adiposity (BMI, BMI-z, WC, and fat mass) and insulin sensitivity, while it tended to have a negative association with β-cell function (*p* = 0.059). Multiple linear regression models for insulin sensitivity and β-cell function can be found in Table 2. MCP-1 and IL-1ra were significant predictors of WBISI, adjusting for age, sex, and fat mass. For every 1 pg/mL increase in MCP-1 and IL-1ra, there was a 0.12 (*p* = 0.027) and 0.03 (*p* = 0.005) unit decrease in WBISI, respectively. Inflammatory mediators were not significantly associated with β-cell function.

Within-group changes and between-group effects on inflammatory mediators and diabetes risk factors after adjusting for age and sex are reported in Table 3. Changes in IL-6, TNF-α, MCP-1, HMW Adpn, IL-10, or IL-1ra were not significantly different between INT and UC. Insulin sensitivity was significantly increased on average in INT by 44.4% (*p* = 0.005) and UC by 75.0% (*p* = 0.002) with no significant differences between groups (interaction, *p* = 0.286). No significant within or between-group effects were noted for β-cell function, BMI, BMI-z, or total fat mass. Body fat percent significantly decreased following INT by 4.0% (*p* < 0.001) compared to 1.5% decrease among UC (*p* = 0.055), which is concordant with the larger trial. Weight significantly increased by 2.3% (*p* = 0.01) and 3.4% (*p* < 0.001) within INT and UC groups, respectively, with no significant differences between groups (interaction, *p* = 0.286). Lean body mass significantly increased by 4.7% in both INT and UC groups (both *p* < 0.001) with no significant differences between them (interaction, *p* = 0.87).

## 4. Discussion

Studies support a reduction in T2D risk factors following lifestyle intervention among high-risk youth [40,41,42], but the mechanisms remain unclear. Our cross-sectional findings suggest that higher levels of MCP-1 and IL-1ra, but not IL-6, TNF-a, HMW Adpn, and IL-10, were significantly associated with decreased insulin sensitivity. Neither of the pro- or anti-inflammatory mediators were significantly associated with β-cell function. However, lifestyle intervention did not lead to any notable changes in either of the pro- and anti-inflammatory mediators examined. These findings add novelty to the body of literature in pediatric obesity by cross-sectionally examining understudied cytokines and chemokines in relation to T2D risk factors, and in response to lifestyle intervention, among an underrepresented and high-risk racial/ethnic population.

Similar to our findings, one other study supported a significant inverse association between MCP-1 and insulin sensitivity [10] among adolescents with obesity while two studies failed to support this association [43,44]. None of these three studies adjusted for age, sex, and adiposity as was conducted in the present study, which may be relevant since the study that showed a significant association between MCP-1 and insulin sensitivity was conducted in older adolescents (~15 y) [10], compared to the studies that found no association which were conducted in younger children [43,44]. In regard to IL-1ra, our results corroborate the findings of two other studies which demonstrated a negative association between IL-1ra and insulin sensitivity [19,20]. IL-1ra is increased in obesity and serves as an anti-inflammatory agent by competing with the same IL-1 receptor on pancreatic β-cells that, when activated, activates a pro-inflammatory cascade [45]. The obesity-induced increase in IL-1ra may serve as a compensatory mechanism in an attempt to protect tissues from obesity-induced inflammation. These cross-sectional data suggest that MCP-1 and IL-1ra may be the key intervention targets for reducing T2D risk factors among Latino youth with obesity.

Of interest is that HMW Adpn was not associated with insulin sensitivity nor β-cell function, which may have been due to low variability among the cohort. Unlike many other cytokines, adiponectin is almost exclusively produced by adipocytes and is an established marker associated with insulin sensitivity and reduced cardiometabolic disease risk among youth [46]. Previous work has demonstrated that low levels of adiponectin among youth predicted future T2D [47] and, among youth who already developed T2D, predicted glycemic failure in response to pharmacotherapies (with or without lifestyle intervention) [48]. Interestingly, HMW Adpn concentrations in our cohort were lower on average than HMW Adpn levels among youth with T2D in another study [48], underscoring the high-risk nature of this cohort of Latino youth with obesity. As for IL-6, TNF-α, and IL-10, their concentrations have varied among pediatric populations in previous studies which may be attributed to the different assays used [31,49,50,51,52,53,54,55,56], age [52], or fitness level [50]. In our sample, IL-10 was very low in concentration (~90% < 1.0 pg/mL), which may be due to obesity, as some other studies have exhibited comparable IL-10 concentrations among youth with obesity compared to normal weight [57,58]. Aside from adiponectin, the literature on adipose tissue related cytokines and chemokines are understudied among high-risk pediatric populations and thus warrant further study of their relationships with T2D risk factors.

In response to lifestyle intervention, our study demonstrated no notable treatment effects on the pro- and anti-inflammatory mediators of interest compared to usual care. Given that the proposed main source of inflammation in obesity is adipose tissue, it is possible that reductions in weight or adiposity, an effect that was not observed in the present study, are needed to induce significant changes in inflammatory mediators. Surgical weight loss studies among youth would support this notion, having demonstrated significant improvements in the pro- and anti-inflammatory milieus following substantial weight loss [59,60,61]. In another study among youth who reduced BMI by an average of 4.5 kg/m^2^ following lifestyle intervention (compared to a 0.2 kg/m^2^ BMI increase in the present study), MCP-1, insulin and other inflammatory mediators were significantly reduced, and adiponectin increased [62]. In high-risk adults, it has been established that 5–7% weight loss is a critical mediator for reducing T2D risk [63] and has led to improvements in adiponectin, IL-6 and TNF-α [5,64,65]. However, although there is evidence in the pediatric literature to suggest that 1.5 kg/m^2^ reductions in BMI are associated with reduced cardiometabolic risk factors [66], physiologic targets are less established in youth as compared to adults. Whether inflammatory mediators generated from adipose tissue can be improved in the absence of weight/adiposity loss among high-risk youth populations warrants further investigation.

There are data to suggest that there is a dose-dependent response of inflammatory markers to exercise. A randomized control trial among older adults showed that adiponectin, and leptin, an appetite-reducing hormone, are increased in response to a resistance training program in an intensity-dependent manner [25]. That study also demonstrated significant reductions in adiposity following resistance training, as measured by BMI [25]. Another study demonstrated that short-term moderate, but not high intensity exercise, led to significant reductions in receptors that are found on macrophages and receive MCP-1 [67]. However, both studies [25,67] included small sample sizes and thus should be considered in the interpretation of results. During acute exercise, IL-6 is known to be released by skeletal muscle with longer duration [68] and higher intensity exercise [69]. This release in IL-6 during acute exercise is thought to stimulate mechanisms that increase anti-inflammatory mediators, IL-10 and IL-1ra [70]. Therefore, it is possible that our lifestyle intervention was not aggressive enough to stimulate anti-inflammatory pathways that work to reduce pro-inflammation.

Quasi-experimental lifestyle intervention studies have demonstrated significant increases in adiponectin [32,33,34,71,72], reductions in IL-6 [73], no changes in IL-10 [74,75], and conflicting results on TNF-α [62,74,75]. Two RCTs failed to support treatment effects on total adiponectin in response to lifestyle intervention compared to control groups, despite significant reductions in weight, adiposity, and C-reactive protein (established marker of systemic inflammation) and increases in insulin sensitivity [76,77]. Another RCT among 15 youth with obesity demonstrated increases in total adiponectin with reductions in IL-6 following a lifestyle intervention that did not induce weight loss but significantly reduced fat mass and improved insulin sensitivity [18,31]. Given the null effects on inflammatory mediators in the present study, increases in insulin sensitivity observed among INT and UC were likely mediated by other factors beyond the markers that were examined. Exercise is important for preventing T2D as skeletal muscle contractions cause glucose uptake through insulin-independent pathways (e.g., AMPK pathways) which may have led to reductions in glucose levels that could have reflected increases in the WBISI and oDI scores [78]. Furthermore, oxidative stress [79], mitochondrial dysfunction [80], free fatty acids [81], myostatin (a protein produced by skeletal muscle) [82], and ectopic fat depots (e.g., liver and visceral fat) [83] have been associated with decreased insulin sensitivity and glucose uptake, and thus changes in these mechanisms may have contributed to the increases in insulin sensitivity. Another study identified leukocytes and neutrophil-leukocyte ratio as predictors of decreased insulin sensitivity and cardiometabolic disease risk [84]. Future work will benefit from rigorous studies that utilize state-of-the-art, high throughput technologies, such as Omics approaches that allow for the examination of hundreds to thousands of genes, proteins, metabolites, and lipid species to understand the underpinning mechanisms by which lifestyle intervention reduces T2D risk factors among high-risk youth.

A strength of this present study is that it is the first to report on obesity-related pro- and anti-inflammatory mediators in response to a lifestyle intervention among youth with prediabetes. β-cell function is an understudied T2D risk factor in relation to obesity-related pro- and anti-inflammatory mediators, unlike insulin sensitivity, thereby representing another strength of this study. Furthermore, this study prioritizes a high-risk Latino youth population that is underrepresented in the field. We acknowledge that this study has some limitations. This analysis only included a sub-sample of a larger RCT and thus was not powered to detect significant changes in the outcomes of interest. The prioritization of Latino youth limits the generalizability of these findings to other youth populations. WBISI and oDI are not the gold standard approaches for measuring insulin sensitivity and β-cell function; however, they have been validated or compared against their respective gold standard in youth with obesity [38,39,85]. Lastly, some data were missing at T2 for inflammatory mediators and T2D risk factors.

## 5. Conclusions

In summary, increased MCP-1 and IL-1ra were predictors of insulin sensitivity, independent of age, sex, and adiposity among Latino youth with obesity and prediabetes. Lifestyle intervention did not lead to relevant changes in pro- and anti-inflammatory markers. Whether changes in these inflammatory markers enhance the reduction of T2D risk factors following lifestyle intervention among high-risk Latino youth may warrant more aggressive interventions. Future rigorous trials are needed to examine the effects of lifestyle intervention on pro- and anti-inflammatory markers and associated changes in insulin sensitivity and β-cell function among high-risk youth populations.

## Figures and Tables

**Table 1:** Baseline characteristics.

Parameter	All (n = 64)	INT (n = 40)	UCC (n = 24)	*p*-Value
Age, y	13.3 ± 1.4	13.4 ± 1.4	13.2 ± 1.4	0.482
Female, n (%)	26 (40.6%)	16 (40.0%)	9 (37.5%)	0.843
BMI, kg/m^2^	32.7 ± 4.8	32.1 ± 3.7	33.7 ± 6.3	0.19
BMI-z	2.2 ± 0.3	2.2 ± 0.3	2.3 ± 0.4	0.275
Fat mass, kg	37.6 ± 9.4	36.2 ± 7.1	40.0 ± 12.2	0.102
WBISI	1.7 ± 1.1	1.8 ± 1.3	1.6 ± 1.0	0.417
oDI	4.5 ± 2.5	4.8 ± 2.5	4.0 ± 2.4	0.15
IL-6, pg/mL	2.3 ± 1.9	1.9 ± 1.7	2.8 ± 2.2	0.091
TNF-α, pg/mL	1.7 ± 0.5	1.7 ± 0.5	1.7 ± 0.4	0.983
MCP-1, pg/mL	644 ± 271	646 ± 250	641 ± 308	0.981
HMW Adpn, ug/mL	1.5 ± 1.7	1.7 ± 0.8	1.3 ± 0.6	0.112
IL-10, pg/mL	0.6 ± 1.0	0.6 ± 1.2	0.6 ± 0.5	0.885
IL-1ra, pg/mL	1150 ± 920	1003 ± 729	1394 ± 1148	0.088

INT: Intervention group; UCC: Usual Care Control group; BMI: body mass index; BMI-z: BMI z-score; WC: waist circumference, A1C: hemoglobin A1C; WBISI: whole-body insulin sensitivity index; oDI: oral disposition index; IL-6: interleukin-6; TNF-α: tumor necrosis factor-alpha; MCP-1: monocyte chemoattractant protein-1; HMW Adpn: high molecular weight adiponectin; IL-10: interleukin-10; IL-1ra: interleukin-1 receptor antagonist.

**Table 2:** Multiple linear regression models: Inflammatory markers as predictors of WBISI or oDI.

	Dependent Variables					
	WBISI			oDI		
Predictors	β	SE	*p*-Value	β	SE	*p*-Value
IL-6	−0.06	0.15	0.677	0.14	0.20	0.488
TNF-α	−0.5	0.5	0.300	1.2	0.8	0.126
MCP-1	−0.12	0.05	**0.027**	−0.013	0.010	0.165
HMW Adpn	0.1	0.1	0.364	0.5	0.4	0.199
IL-10	0.3	0.4	0.786	−0.5	0.4	0.200
IL-1ra	−0.03	0.01	**0.005**	−0.007	0.004	0.074

Controlled for age, sex, and fat mass; Alpha level set at 0.05; Significant associations are bolded.

**Table 3:** Changes in biomarkers within and between INT and UC groups from T1 to T2.

	UC (n = 24)			INT (n = 40)			Treatment Effect
Parameters	T1	T2	Within-Group Effect *p*-Value	T1	T2	Within-Group Effect *p*-Value	ΔT2-T1 (95%CI) *p*-Value
IL-6	2.8 ± 0.4	2.4 ± 0.2	0.236	1.9 ± 0.3	1.8 ± 0.2	0.675	0.2 (−0.6, 1.1) 0.574
TNF-α	1.7 ± 0.1	1.7 ± 0.1	0.926	1.7 ± 0.1	1.4 ± 0.1	0.067	−0.1 (−0.3, 0.1) 0.223
MCP-1	641 ± 51	603 ± 49	0.167	646 ± 36	914 ± 228	0.239	30.7 (−143.3, 756.9) 0.182
HMW Adpn	1.28 ± 0.11	1.21 ± 0.11	0.303	1.67 ± 0.12	1.42 ± 0.11	**0.001**	−0.18 (−0.37, 0.02) 0.079
IL-10	0.6 ± 0.11	0.5 ± 0.09	0.496	0.6 ± 0.18	0.3 ± 0.03	0.096	−0.2 (−0.7, 0.2) 0.351
IL-1ra	1394 ± 219	1230 ± 193	0.235	1003 ± 110	843 ± 95	0.086	0.4 (−325.3, 326.1) 0.981
WBISI	1.6 ± 0.2	2.8 ± 0.5	**0.002**	1.8 ± 0.2	2.6 ± 0.4	**0.005**	−0.4 (−1.4, 0.5) 0.352
oDI	4.0 ± 0.5	3.9 ± 0.5	0.781	4.8 ± 0.4	5.4 ± 0.5	0.323	0.7 (−0.7, 2.1) 0.345
Weight	89 ± 4	92 ± 4	**<0.001**	86 ± 2	88 ± 2	**0.006**	−1.3 (−3.2, 0.5) 0.143
BMI	33.7 ± 1.1	34.0 ± 1.2	0.200	32.1 ± 0.5	32.1 ± 0.6	0.805	−0.3 (−0.9, 0.4) 0.426
BMI-z	2.28 ± 0.07	2.24 ± 0.09	0.245	2.19 ± 0.04	2.15 ± 0.05	0.059	−0.009 (−0.07, 0.68) 0.796
Fat Mass	40.0 ± 2.3	40.5 ± 2.4	0.393	36.2 ± 1.0	35.3 ± 1.1	0.094	−1.4 (−2.9, 0.2) 0.085
Body Fat %	47.0 ± 0.9	46.3 ± 1.0	0.055	44.5 ± 0.6	42.7 ± 0.7	**<0.001**	**−1.0 (0.02, 2.078)** **0.045**
Lean Mass	42 ± 1	44 ± 1	**<0.001**	43 ± 1	45 ± 1	**<0.001**	0.1 (−0.08, 0.06) 0.840

Alpha level set at 0.05; Significant *p*-values are bolded; Data under T1 and T2 columns are presented as FIML-adjusted Mean ± SE. Treatment Effect column displays FIML-adjusted differences in changes (meanΔ ± 95%CI) in outcomes (ΔINT − ΔUC). Missing data at T2: N = 1 (MCP-1, Fat Mass, Lean Mass, Body Fat%); N = 2 (IL-6, TNF-α); N = 7 (WBISI); N = 8 (oDI).

## Data Availability

At the time of the study, informed consent was not obtained from study participants to make data publicly available.

## Supplementary Materials

The following supporting information can be downloaded at: https://www.mdpi.com/article/10.3390/nu15112442/s1. Table S1: Baseline correlations: inflammatory markers, adiposity and T2D risk factors (n = 64).
