# Supplementary material for: Inflammatory Mediators and Type 2 Diabetes Risk Factors before and in Response to Lifestyle Intervention among Latino Adolescents with Obesity
Source: Nutrients. 2023 May 24;15(11):2442. doi: 10.3390/nu15112442 (PMC10255740; doi:10.3390/nu15112442)
Supplement: Supplementary file 1 [file nutrients-15-02442-s001.zip › nutrients-2366583-supplementary.pdf]

Table S1. Baseline correlations: inflammatory markers, adiposity and T2D risk factors (n=64)

		Age	BMI	BMI-z	WC	Fat Mass	WBISI	oDI
IL-6	*r*	-0.224	**0.293**	**0.327**	0.167	0.221	-0.160	-0.202
	p	0.075	**0.019**	**0.008**	0.186	0.080	0.207	0.109
TNF-α	*r*	-0.206	0.127	**0.295**	0.190	0.173	-0.121	0.104
	p	0.102	0.318	**0.018**	0.133	0.171	0.341	0.412
MCP-1	*r*	-0.063	0.090	**0.278**	0.207	0.084	-0.217	-0.155
	p	0.619	0.480	**0.026**	0.102	0.512	0.084	0.222
HMW Adpn	*r*	-0.016	-0.083	-0.076	-0.205	-0.142	0.082	0.215
	p	0.901	0.517	0.553	0.105	0.264	0.518	0.087
IL-10	*r*	-0.094	-0.026	0.028	-0.123	-0.117	0.066	-0.148
	p	0.461	0.838	0.828	0.334	0.359	0.606	0.242
IL-1ra	*r*	0.197	**0.300**	**0.339**	**0.297**	**0.262**	**-0.309**	-0.237
	p	0.119	**0.016**	**0.006**	**0.017**	**0.036**	**0.013**	0.059

Alpha level is set at 0.05; Significant correlations are bolded
r = correlation coefficient; p = p-value
